# Eco-Conscious Approach to Thermoresponsive Star-Comb and Mikto-Arm Polymers via Enzymatically Assisted Atom Transfer Radical Polymerization Followed by Ring-Opening Polymerization

**DOI:** 10.3390/molecules29010055

**Published:** 2023-12-21

**Authors:** Tomasz Fronczyk, Anna Mielańczyk, Olesya Klymenko, Karol Erfurt, Dorota Neugebauer

**Affiliations:** 1 Department of Physical Chemistry and Technology of Polymers, Faculty of Chemistry, Silesian University of Technology, M. Strzody 9 Street, 44-100 Gliwice, Poland; tomasz.fronczyk@polsl.pl (T.F.); dorota.neugebauer@polsl.pl (D.N.); 2 Department of Histology and Cell Pathology, Faculty of Medical Sciences in Zabrze, Medical University of Silesia, 40-055 Katowice, Poland; oklymenko@sum.edu.pl; 3 Department of Organic Chemical Technology and Petrochemistry, Faculty of Chemistry, Silesian University of Technology, B. Krzywoustego 4, 44-100 Gliwice, Poland; karol.erfurt@polsl.pl

**Keywords:** star-comb polymers, stimuli-responsive polymers, ATRP, enzymes

## Abstract

This study explores the synthesis, characterization, and application of a heterofunctional initiator derived from 2-hydroxypropyl cyclodextrin (HP-β-CD), having eight bromoester groups and thirteen hydroxyl groups allowing the synthesis of mikto-arm star-shaped polymers. The bromoesterification of HP-β-CD was achieved using α-bromoisobutyryl bromide as the acylation reagent, modifying the cyclodextrin (CD) molecule as confirmed by electrospray ionization mass spectrometry (ESI-MS), nuclear magnetic resonance (NMR), attenuated total reflection-Fourier transform infrared (ATR-FTIR) spectroscopy analysis, and differential scanning calorimetry (DSC) thermograms. The initiator’s effectiveness was further demonstrated by obtaining star-comb and mikto-arm polymers via an enzymatically assisted atom transfer radical polymerization (ATRP) method and subsequent ring-opening polymerization (ROP). The ATR polymerization quality and control depended on the type of monomer and was optimized by the way of introducing the initiator into the reaction mixture. In the case of ATRP, high conversion rates for poly(ethylene oxide) methyl ether methacrylate (OEOMA), with molecular weights (Mn) of 500 g/mol and 300 g/mol, were achieved. The molecular weight distribution of the obtained polymers remained in the range of 1.23–1.75. The obtained star-comb polymers were characterized by different arm lengths. Unreacted hydroxyl groups in the core of exemplary star-comb polymers were utilized in the ROP of ε-caprolactone (CL) to obtain a hydrophilic mikto-arm polymer. Cloud point temperature (T_CP_) values of the synthesized polymers increased with arm length, indicating the polymers’ reduced hydrophobicity and enhanced solvation by water. Atomic force microscopy (AFM) analysis revealed the ability of the star-comb polymers to create fractals. The study elucidates advancements in the synthesis and utilization of hydrophilic sugar-based initiators for enzymatically assisted ATRP in an aqueous solution for obtaining complex star-comb polymers in a controlled manner.

## 1. Introduction

Atom transfer radical polymerization (ATRP) is one of the most commonly used methods of reversible-deactivation radical polymerization, which produces a wide range of polymers with strictly defined parameters, such as molecular weight, chain length, topology, and side and end group functionalities. Currently, researchers are seeking environmentally friendly methods of performing ATRP following the principles of green chemistry [1,2,3]. Among them are the ATRP methods that allow the reaction to proceed in the presence of oxygen, including photo-induced ATRP [4], initiators for continuous activator regeneration (ICAR) ATRP [5], and enzymatically assisted ATRP [6,7,8,9]. On the other hand, the shift of ATRP toward green chemistry is also possible due to the usage of initiators based on natural compounds such as carbohydrates, including monosaccharides, disaccharides, oligosaccharides, and polysaccharides [10,11]. Cyclodextrins (CDs) are cyclic oligosaccharides composed of α-D-glucose units linked by α-1,4-acetal bonds. The most widely studied variants of CDs are α-, β-, and γ-CDs, which contain 6, 7, or 8 α-D-glucose units, respectively. CDs are toroidal in shape, with all primary hydroxyl (-OH) groups located on the narrow side and the secondary -OH groups on the wider side. The toroidal shape makes CDs hydrophilic on the outside, though the cavity stays hydrophobic [12,13,14]. CDs are used as excipients in several FDA-approved pharmaceutical products, such as liposomal formulations of doxorubicin, daunorubicin, cytarabine, and amphotericin B [15]. Hydroxypropyl-β-CD is an FDA-approved compound for solubilizing, capturing, and delivering lipophilic drugs in humans, and it has been shown to reverse atherosclerosis in preclinical studies [16]. The use of CD allows the synthesis of star polymers, which are a type of branched polymers. They are characterized by linear or comb-like arms extending from a centrally located core [17]. Star polymers owe their advantageous properties to their unique topological structure. Due to the smaller number of entanglements of the arms, star-shaped polymers have a lower solution viscosity in dilute solutions, compared to analogous linear polymers of the same molecular weight. In addition, polymer viscosity and elasticity change with the length of the arms [18]. Star polymers show remarkable stimuli-responsiveness due to the high density of functional groups that may be present in their structure [19].

Star polymers show great promise in the field of biomedical applications, particularly controlled-release drug delivery systems [20,21,22,23,24]. Moreover, star polymers have been found to play a significant role in the crystallization processes. They can be used to control the nucleation and growth of crystals, which can lead to the formation of unique crystal structures. This has potential applications in various fields, including drug delivery, where the controlled crystallization of therapeutic compounds can affect their bioavailability and efficacy [25]. Star polymers can also remove heavy metals from water, which is related to the star polymer’s spatial arrangement. For example, the tetragonal star-like polyaniline microstructure was applied to selectively and quickly remove Cu(II) and Pb(II) from water [26].

This article presents the results of the synthesis and utilization of bromoisobutyryl-functionalized bio-safe CD derivatives as ATRP initiators, based on (2-hydroxypropyl)-β-CD (HP-β-CD). A heterofunctional initiator with eight bromoester groups (8-Br-HP-β-CD) was used in an enzymatically assisted ATRP (glucose oxidase), also known as breathing ATRP, to obtain 8-arm star-comb poly(oligo ethylene oxide))methyl ether methacrylate (POEOMA) from OEOMA500 and OEOMA300. Subsequently, a mikto-arm star-comb polymer (MSCP1) with additional poly(ε-caprolactone) arms was obtained based on the star-shaped polymer with eight POEOMA500 arms.

## 2. Results and Discussion

**Figure 1:**
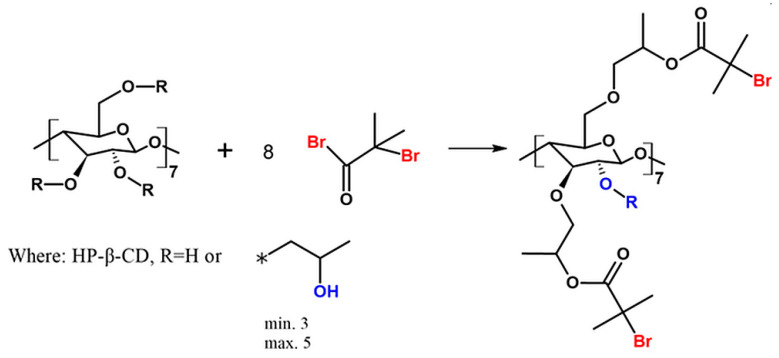
Scheme of the synthesis of heterofunctional initiator based on CD. The asterisk (*) indicates the point of connection of the 2-hydroxypropyl group instead of R to the main molecule.

The heterofunctional initiator based on HP-β-CD was synthesized and characterized (Figure 1).

Multifunctional initiators based on β-cyclodextrin are described in the literature [27,28,29]. Admittedly, an ATRP initiator with one bromoester group based on HP-β-CD was previously described by Zhu et al. [30]. However, this is the first time when HP-β-CD initiator with eight bromoester groups has been synthesized according to the slightly changed procedure described by Pan et al. (utilized for β-CD) [31]. In this paper, we describe for the first time the utilization of CD-based initiator in enzymatically assisted ATRP as an effective route to obtain star-comb and mikto-arm star-comb polymers.

**Figure 2:**
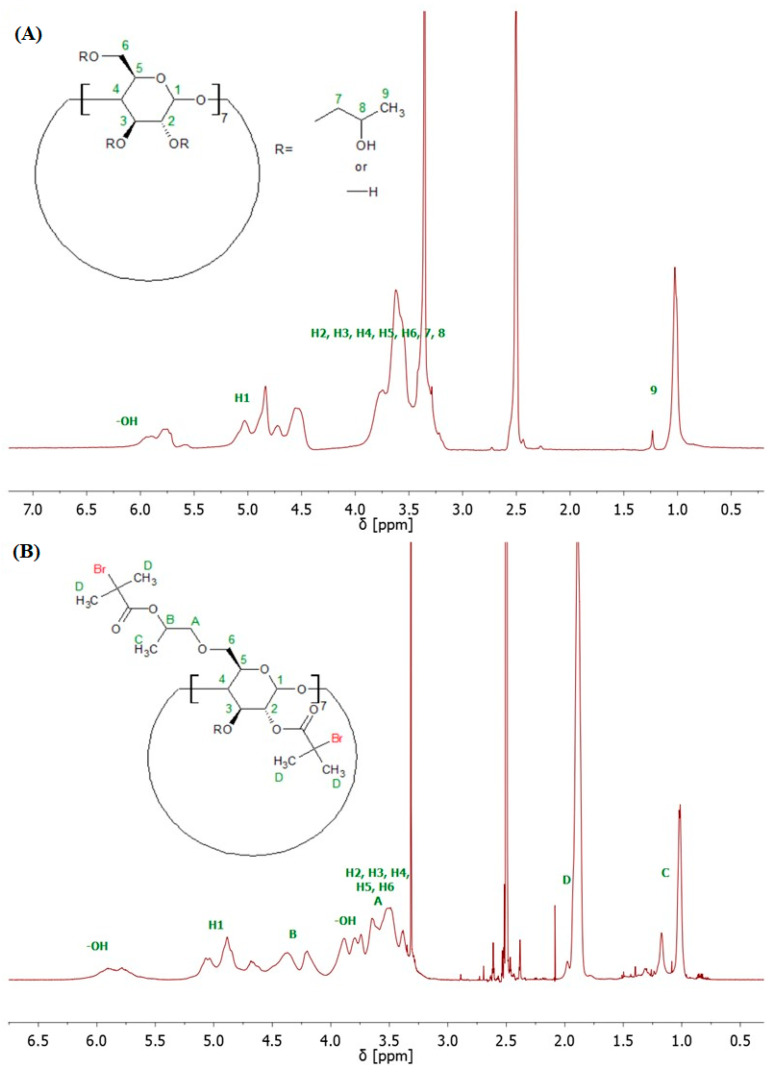
^1^H NMR (600 MHz, DMSO-d_6_) spectra of HP-β-CD (**A**), and 8-Br-HP-β-CD (**B**).

The structure of 8-Br-HP-β-CD was confirmed by spectroscopic and spectrometric analyses. ATR-FTIR analysis confirmed the bromoesterification reaction (Supplementary Figure S2). The peak corresponding to the stretching vibrations of the -OH group decreased its intensity after the reaction, which indicates partial substitution of the -OH groups in the molecule. The peak responsible for the –C=O stretching vibrations present in the spectrum of the initiator proves that the CD was successfully modified. Analysis of the ^1^H NMR spectrum confirmed the presence of eight bromoester groups in the CD molecule (Figure 2). The degree of -OH groups substitution in HP-β-CD was calculated from the integrals of the peaks assigned to the acetal proton (H1) and six protons from the bromoester group.

**Figure 3:**
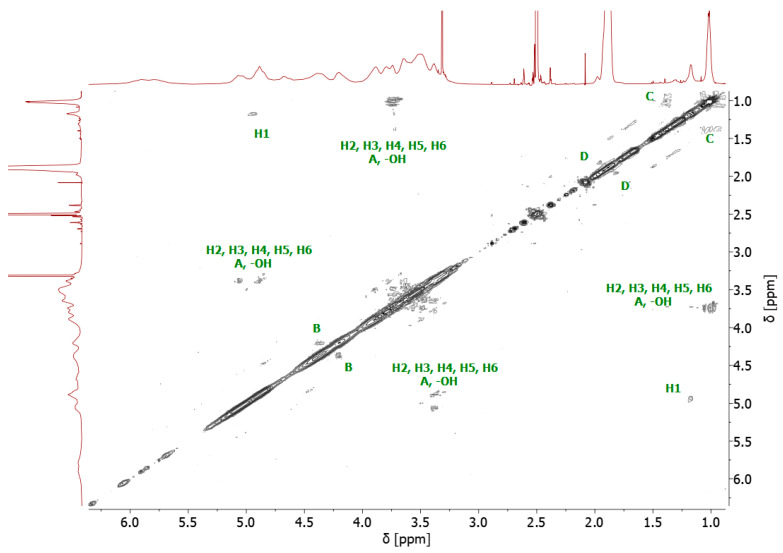
^1^H-^1^H homonuclear gCOSY 2D NMR spectrum of 8-Br-HP-β-CD.

The bromoesterification of the initiator with the -OH groups was also verified by ^13^C NMR spectroscopy. The ^13^C NMR spectrum of the bromoesterified initiator exhibited a signal at 171 ppm, corresponding to the carbonyl carbon of the ester group, and a signal at 31 ppm, corresponding to the carbon attached to the bromine atom (Supplementary Figure S3). Two-dimensional (2D) gradient-selected correlation spectroscopy (gCOSY) NMR was also performed to confirm the structure and corresponding signals (Figure 3).

DSC thermograms showed that the esterification of CDs affects the thermal properties of the obtained bromoester derivatives, changing the melting temperature from 219.67 °C (HP-β-CD) to 210.23 °C (8-Br-HP-β-CD) (Supplementary Figure S4).

**Figure 4:**
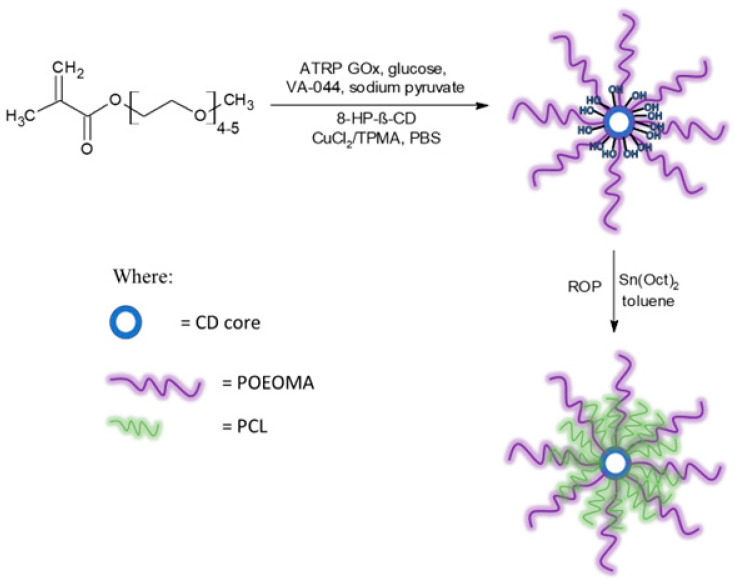
Reaction scheme for the synthesis of star-comb polymers and mikto-arm star-comb polymers.

Next, 8-Br-HP-β-CD was used to obtain star-comb polymers via an enzymatically assisted ATRP method using a CuCl_2_/TPMA catalyst complex in phosphate-buffered saline, pH 7.4. Each reaction was carried out for 2 h at 45 °C (Figure 4).

**Figure 5:**
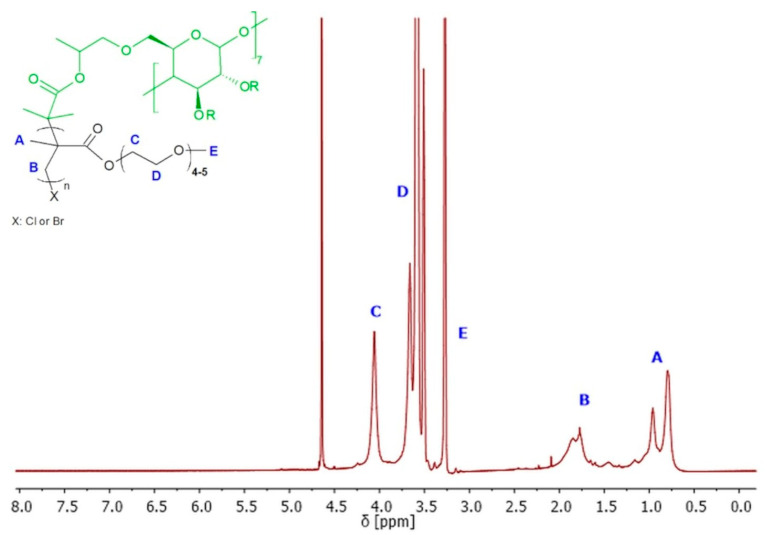
^1^H NMR (600 MHz, D_2_O) spectra of exemplary 8-arm star-comb polymer SCP7.

Polymers SCP1–SCP3 were obtained with 91–94% of monomer conversion, while polymers SCP4–SCP9 were obtained with 65–89% of monomer conversion (Table 1). The ^1^H NMR spectroscopy was used to confirm the successful synthesis and purification of the polymer, as well as to determine the conversions of monomers, the average degree of polymerization (DP), and finally theoretical number average molecular weight (M_n_). The ^1^H NMR spectrum of the polymer showed characteristic signals for the repeating units, and no signals for the impurities or the unreacted monomer (Figure 5).

**Figure 6:**
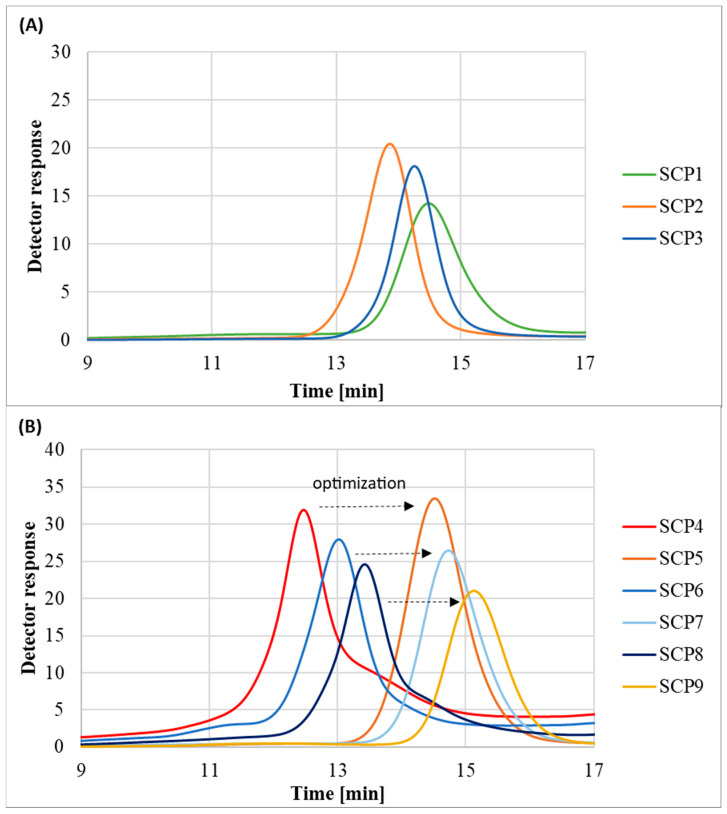
SEC traces of eight-arm POEOMA star-comb polymers: (**A**) POEOMA500 polymers; (**B**) POEOMA300 polymers.

The molecular weight distribution values of the obtained polymers SCP1-SCP3 stayed in the range of 1.23–1.38 and chromatograms showed monomodal peaks, which indicates that the OEOMA500-based polymers were synthesized in a controlled manner (Figure 6A). However, in the case of OEOMA300-based polymers synthesized without the addition of methanol, it was observed that besides the main signal, there were also peaks corresponding to the high and low molecular weight species. In order to regain control over the polymerization process, the reactions were repeated with the exception that the initiator was dissolved in a small amount of methanol prior to addition to the reaction mixture. Lack of control over the polymerization process in the case of the OEOMA300 monomer was probably linked with its lower hydrophilicity in comparison to the OEOMA500 (Figure 6B) [32].

The polymers prepared with the initiator dissolved in methanol had lower Đ than the polymers prepared with the solid initiator, indicating a more homogeneous and efficient initiation process (Figure 6B). However, the dissolution of the initiator in methanol also slightly reduced the monomer conversion, suggesting a trade-off between dispersity and yield. This can be explained by the fact that the activity of enzymes like glucose oxidase can be significantly influenced by the solvent environment [33]. Enzymes, being proteins, have a specific three-dimensional structure crucial for their activity. For instance, organic solvents, including alcohols like ethanol or methanol, can cause changes in the protein structure, often leading to decreasing activity, denaturation, or inactivation of the enzyme [34].

**Figure 7:**
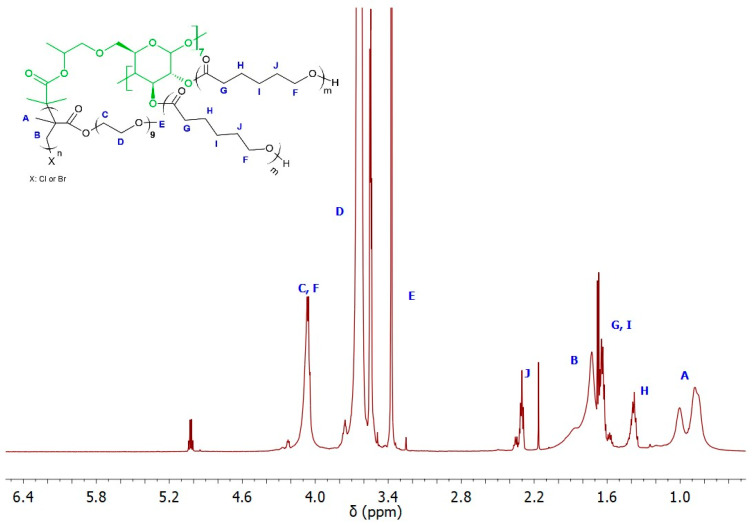
^1^H NMR (600 MHz, CDCl_3_) spectra of MSCP1.

The ability of the remaining hydroxyl groups in the core of the star-comb macromolecules to initiate polymerization was examined by performing ROP of ε-CL. As model macroinitiators, SCP3 and SCP9 were selected due to the lowest molecular weight from both groups of star-comb polymers with POEOMA300 and POEOMA500 arms. However, only a mikto-arm star-comb polymer based on SCP3 retained solubility in water, which had a crucial impact on its further directions of application. Figure 7 shows the spectrum of MSCP1 where characteristic signals from polymethacrylate and polyester arms can be seen. The mole fractions of PCL arms in MSCP1 and MSCP2 were calculated from the NMR spectra, based on the integral values from the signal at 1.4 ppm (marked on the spectrum as H) and methyl groups in the range from 0.5 to 1.2 ppm (marked on the spectrum as A), and were equal to 21% and 36%, respectively.

The conversion of the ε-CL was equal to 36% for MSCP1 and 69% for MSCP2, and the obtained mikto-arm polymer exhibited decreased M_n, SEC_ value in comparison to the macroinitiator (for MSCP1 51,300 g/mol vs. 103,100 g/mol and for MSCP2 29,100 g/mol vs. 34,400 g/mol). However, in the case of the dispersity index, comparing the macroinitiator to the polymer, the results are as follows: Đ_MSCP1_ = 1.20 vs. Đ_SCP3_ = 1.23 and for MSCP2 Đ_MSCP2_ = 1.31 vs. Đ_SCP3_ = 1.27 (Supplementary Figure S5 and Table S1).

**Figure 8:**
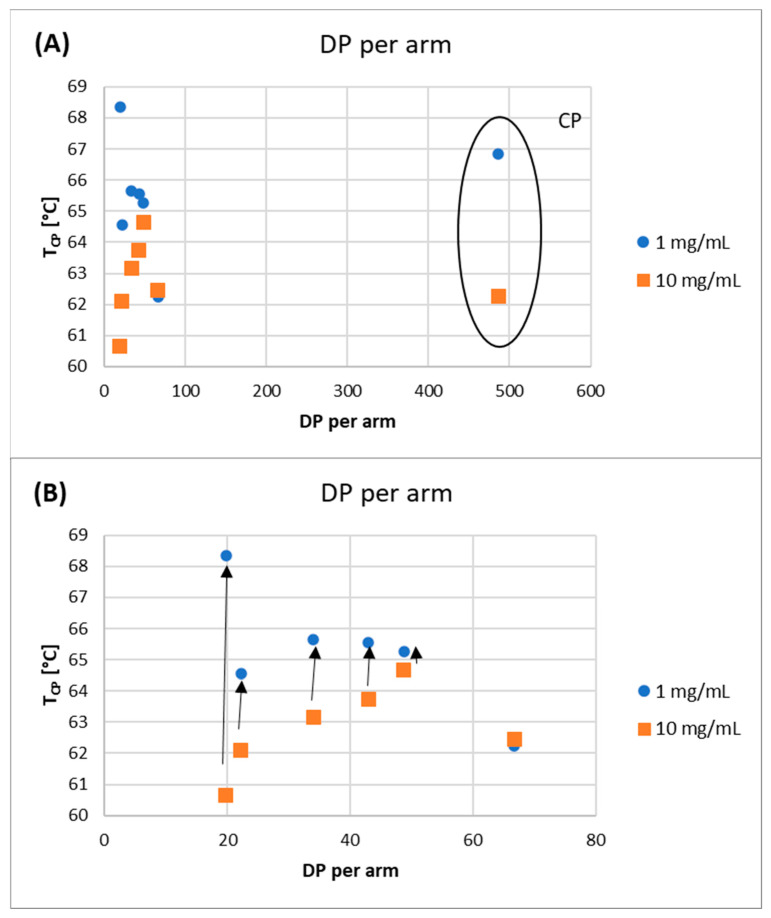
T_CP_ as a function of DP per arm of obtained polymers: (**A**) All polymers, (**B**) a subset of the top graph showing only star-comb polymers.

The studies of the phase transition of the polymer solutions showed good reproducibility and reversibility, as evidenced by the almost identical T_CP_ values on the first and second heating curves. The T_CP_ values of the polymer solutions decreased with increasing polymer concentration (Figure 8).

**Figure 9:**
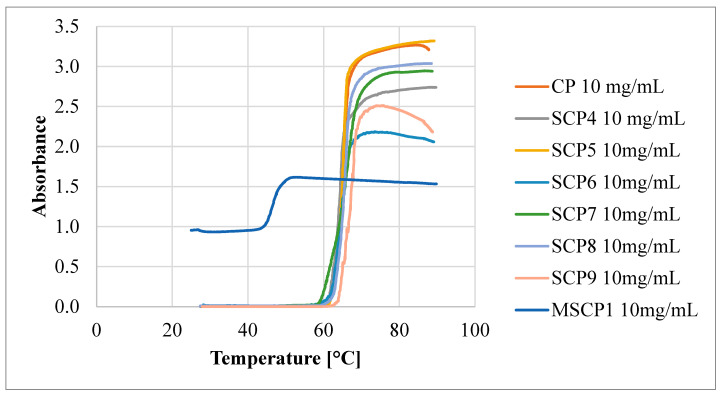
Absorbance as a function of the temperature of obtained polymers.

This indicates that the polymer–polymer interactions are stronger than the polymer–water interactions at higher polymer concentrations. However, as the DP_arm_ values increase, the differences between the T_CP_ values decrease, until the DP_arm_ > 60 repeating units, where this difference disappears completely. To verify the effect of polymer topology on the T_CP_ value, a comb-like polymer (CP) was synthesized via the utilization of HO-EBiB initiator and OEOMA300 as the monomer. The CP obtained using the enzymatically assisted ATRP method displayed T_CP_ values comparable to those reported in the literature. Namely, T_CP_ of P(OEOMA_300_) solution in water at a concentration of 2 mg/mL was equal to 66.5 °C, and for P(OEOMA_300_) solution in water at a concentration of 5 mg/mL T_CP_ was equal to 66 °C [35,36]. In our studies, T_CP_ values for CP solution in water were equal to 66.85 °C at a concentration of 1 mg/mL and 62.25 °C at a concentration of 10 mg/mL These results slightly differed from those obtained for the star-comb polymers (T_CP_ in the range of 60.65–64.65 °C for 10 mg/mL), confirming that the polymer architecture influences the T_CP_. This evidence further supports the notion that the star-comb architecture does indeed affect the thermosensitivity of the polymers. In general, the T_CP_ values of the star-comb polymers showed a positive correlation with the arm length, i.e., the degree of polymerization per arm. It can be explained by the reduced hydrophobicity and enhanced solvation of the polymers with longer arms. The absorbance plots of the tested polymers differ in terms of maximum end values (Figure 9).

This may be related to the topology of the tested compounds, which makes it easier to form clusters of polymers. Different absorbance values may also be related to different DP per arm of the tested polymers. A star-comb polymer with short arms may adopt a more “tight” conformation where the arms are close together. On the other hand, a polymer with longer arms may adopt a more “spread out” conformation where the arms are far apart. These different conformations can affect the availability and exposure of the functional groups that can absorb light.

In the experimental evaluation of polymers designated as SCP1-SCP3, the absence of a T_CP_ was observed, corroborating their hydrophilic characteristics. Specifically, the absorbance levels measured via UV–Vis spectroscopy demonstrated negligible variations, thereby substantiating the hydrophilic nature of these polymers. Conversely, the mikto-arm star-comb polymer synthesized from poly(oligo(ethylene glycol) methyl ether methacrylate) (POEOMA500) and ε-caprolactone (SCP3) exhibited T_CP_ in water. At a concentration of 1 mg/mL, the T_CP_ was equal to 47.65 °C, while at a 10 mg/mL concentration, the T_CP_ decreased to 45.35 °C. These data suggest that the incorporation of poly(ε-caprolactone) (PCL) arms changed the hydrophilic/hydrophobic balance, enabling the phase transition of mikto-arm star-comb polymer in water solution.

**Figure 10:**
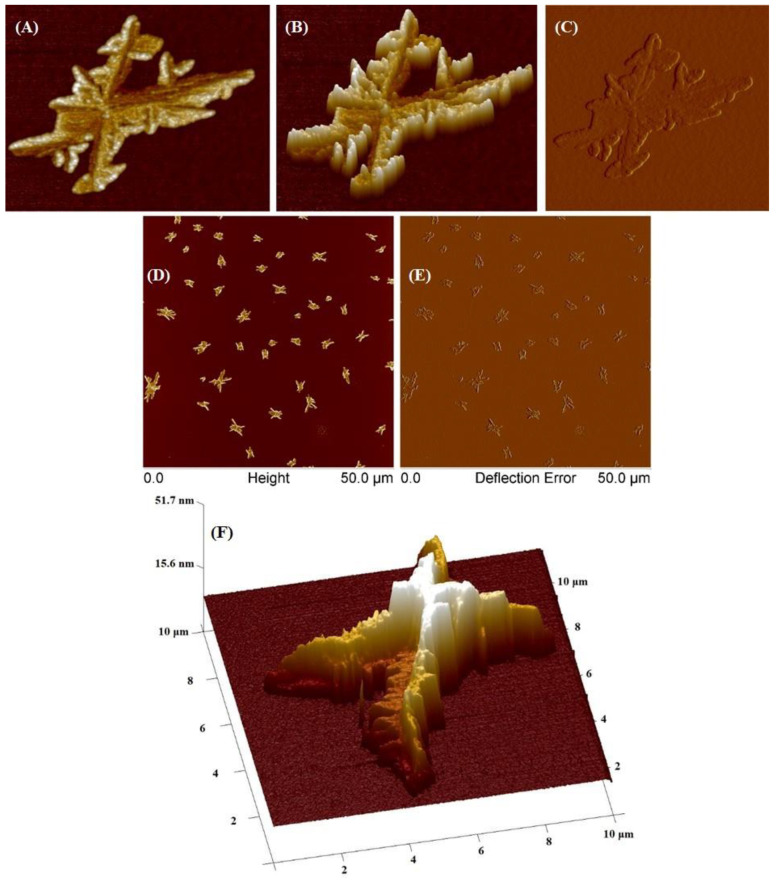
AFM images of obtained star-comb-polymer SCP2 at a concentration of 0.01 g/mL. (**A**–**C**) images of a single structure, (**D**,**E**) images of various structures, (**F**) 3D image of a single structure.

The SCP2 polymer was investigated utilizing AFM (Figure 10). At a concentration of 0.01 mg/mL, SCP2 formed large aggregates and individual fractal-like elements upon drying of the substrate. The average height of these individual objects was 19 nm, with a minimum height of 10 nm and a maximum of 50 nm. The average surface area of stars in the sample is 2.3 μm² (with a range of 0.5–8 μm²), and the average diameter was 1.7 μm (with a range of 0.81–3.3 μm). Moreover, large aggregates with a diameter of ~10 μm were also detected, but these were isolated cases. The formation of aggregates with sizes around 10 μm is most likely related to the stronger intermolecular interaction between macromolecules than the macromolecule–substrate interaction. It is worth noticing that this phenomenon was also observed and described previously in the literature by other researchers who studied crystal patterns formed by polyethylene oxide (PEO) [37].

Despite its low concentration of 0.001, the SCP2 sample formed single aggregates of various geometric shapes on the surface of the polymer mica (Supplementary Figure S6). These aggregates had diameters ranging from 50 to 60 μm and heights between 300 and 500 nm.

**Figure 11:**
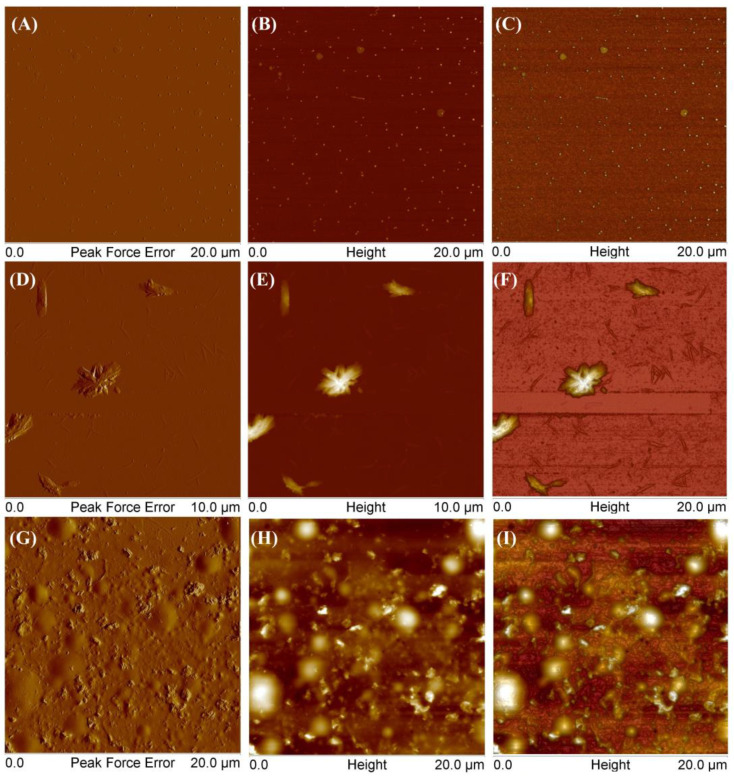
AFM images of obtained star-comb-polymer SCP3 at a concentration of 0.001 g/mL (**A**–**F**) and at a concentration of 0.01 g/mL (**G**–**I**).

To compare polymers obtained from different monomers, SCP3 was also examined (Figure 11).

**Figure 12:**
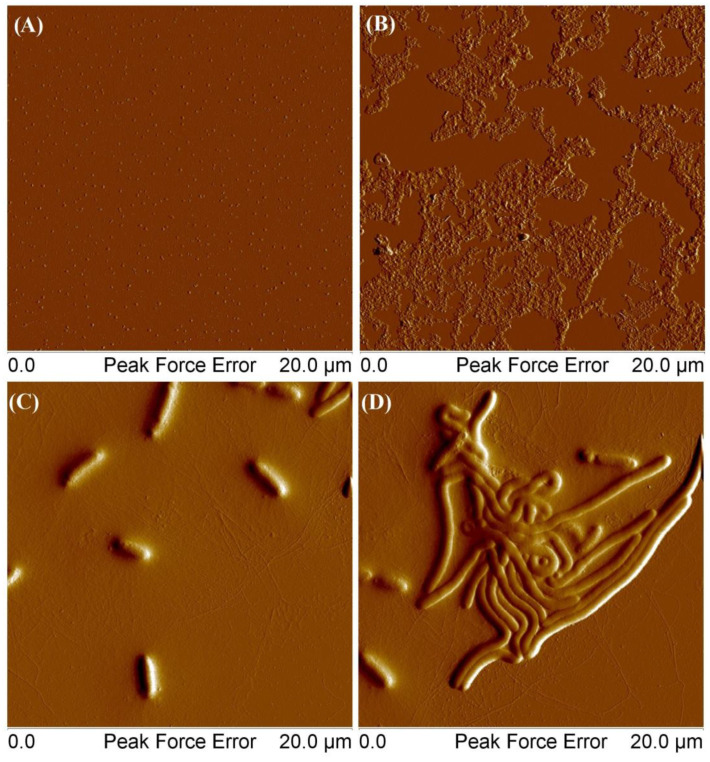
AFM images of obtained MSCP1: At a concentration of 0.001 g/mL (**A**), at a concentration of 0.01 g/mL (**B**), and at a concentration of 0.001 g/mL from a solution that was set aside for a week (**C**,**D**).

At the lowest tested concentration, the polymer exhibited several distinct forms. Figure 11A–C show round aggregates with a height of 10 to 17 nm and a diameter of 120 to 250 nm. Additionally, the polymer also formed larger aggregates with thicknesses equal to 4–6 nm and the diameter in the range 300–610 nm. Other scans (D–F) showed flat, elongated objects with a thickness of 5–7 nm and a length of 95–260 nm and higher branched ones with a thickness of 500–900 nm and a length of 1.39–2.21 μm. As the concentration increased, the polymer tended to form a coating on the mica surface with round convexes with a height of 180–300 nm (G–I). Figure 12 presents AFM images of mikto-arm polymers MSCP1.

MSCP1 at a concentration of 0.001 mg/mL formed single clusters with an average height of 8 nm. For a concentration of 0.01 mg/mL, the thickness of the deposited layer ranged from 30 to 70 nm. However, in the case of polymers deposited from solutions that were left to stand for a week (picture C), the average height of the structures was 270 nm, the length ranged from 1.85 μm to 3.31 μm, and the height of thin fibrils stayed within the range 1–3 nm. For picture D, the height of large elements was 130–170 nm, and the height of thin filaments was between 1 and 3 nm. The self-organization of the star polymer comprising a cyclodextrin core, 8 arms of POEOMA500, and 13 arms of polycaprolactone in an aqueous environment could be influenced by the amphiphilic nature of the polymer and the specific properties of its components. The hydrophilic POEOMA500 arms could possibly extend into the aqueous medium, while the hydrophobic polycaprolactone arms might avoid water, potentially leading to an asymmetric structure that could facilitate self-organization over time. The formation of wormlike micelles depends on the ratio between the hydrophilic and hydrophobic segment of the mikto-arm polymer, and it is initiated by the spontaneous collapse of the water-insoluble phase, large enough to form a micellar core. Presumably, the entanglements of hydrophobic chains facilitated the connection of many individual macromolecules to form a cylindrical core, while hydrophilic chains surrounding the core stabilized the cylindrycal structure. Our observation aligns with the documented behaviors of amphiphilic block copolymers possessing different macromolecular architectures [38,39]. In such systems, the interplay between hydrophilic and hydrophobic interactions is finely balanced, leading to the self-assembly of various morphologies, including spheres, rods, and vesicles, depending on the copolymer’s composition and the surrounding conditions. This process is significantly influenced by the thermodynamics of the system, where factors like temperature, solvent quality, and polymer concentration play decisive roles in determining the final structure of the self-assembled micelles.

Based on the AFM images, polymeric aggregates exhibit an irregular, fractal-like morphology, suggesting a semi-crystalline polymer, which was confirmed by DSC analysis (Supplementary Figure S7) [40,41]. They are not flat, exhibiting variations in height across their entire surface, which indicates a complex three-dimensional structure. The surface topology of the imaged aggregates is characterized by numerous irregularities and protrusions, possibly due to the unique molecular structure of the individual macromolecules.

## 3. Materials and Methods

HP-β-CD (ThermoFisher, 97%, Waltham, MA, USA), 4-(N, N-dimethylamino)-pyridine (DMAP, Sigma-Aldrich, ≥99%, Poznan, Poland), triethylamine (TEA, Sigma-Aldrich, ≥99.5%, Poznan, Poland), anhydrous N-methyl-2-pyrrolidione (NMP, Sigma-Aldrich, 99.5%, Poznan, Poland), 2-bromoisobutyryl bromide (BiBB, Sigma-Aldrich, 98%, Poznan, Poland), 2-hydroxyethyl 2-bromoisobutyrate (HO-EBiB, Sigma-Aldrich, 95%, Poznan, Poland), tin(II) 2-ethylhexanoate (Alfa Aesar, 96%, Warsaw, Poland ), poly(ethylene glycol) methyl ether methacrylate (OEOMA300, Sigma-Aldrich, Poznan, Poland), poly(ethylene oxide) methyl ether methacrylate (OEOMA500, Sigma-Aldrich, Poznan, Poland), ε-caprolactone (Alfa Aesar, 99%, Warsaw, Poland), 2,2′-azobis [2-(2-imidazolin-2-yl)propane] dihydrochloride (VA-044, TCI, >98%, Eschborn, Germany), glucose (TCI, 98%, Eschborn, Germany), glucose oxidase (GOx) (Type XS, Sigma-Aldrich, Poznan, Poland), copper (II) chloride CuCl_2_ (Sigma-Aldrich, 99.995%, Poznan, Poland), tris(2-pyridylmethyl)amine (TPMA, Sigma-Aldrich, >98%, Poznan, Poland), sodium pyruvate (TCI, >97%, Eschborn, Germany), cyclohexane (Chempur, 99.5%, Piekary Slaskie, Poland), tetrahydrofuran (THF) (EUROCHEM BGD, p.a., Tarnow, Poland), methanol (Chempur, p.a., Piekary Slaskie, Poland), dimethylformamide (Chempur, p.a., Piekary Slaskie, Poland), n-heptane (Chempur, p.a., Piekary Slaskie, Poland), toluene (STANLAB, p.a., Lublin, Poland), chlorotrimethylsilane (Sigma-Aldrich, ≥98%, Poznan, Poland). All chemicals were used as received.

An amount of 0.01 M Phosphate buffer saline (PBS, pH 7.4, Sigma-Aldrich, Poznan, Poland) was prepared according to the following procedure: one tablet was dissolved in 200 mL of deionized water yielding 0.01 M phosphate buffer saline (0.0027 M potassium chloride and 0.137 M sodium chloride).

## 4. Instrumentation

### 4.1. Nuclear Magnetic Resonance (NMR)

^1^H NMR and ^13^C NMR spectra of the synthesized initiator, polymers, and reaction mixtures were collected on a Varian Inova 600 MHz spectrometer (Palo Alto, CA, USA) at 25 °C using DMSO-d_6_ as a solvent and tetramethylsilane (TMS) as an internal standard for initiator; deuterium oxide (D_2_O) for OEOMA-based homopolymers and corresponding reaction mixtures; deuterated chloroform (CDCl_3_) for MSCP1 and MSCP2, and corresponding reaction mixtures. The samples were prepared by dissolving ~7 mg of the solid samples in 0.7 cm^3^ of deuterated solvent, and in the case of reaction mixtures 0.12 cm^3^ (0.01 cm^3^ for MSCP1-2) of the reaction mixture in 0.58 cm^3^ of deuterated solvent.

### 4.2. Attenuated Total Reflectance-Fourier Transform Infrared Spectroscopy (ATR-FTIR)

ATR-FTIR spectra were recorded on a Perkin-Elmer Spectrum Two 1000 FT-IR Infrared Spectrometer with an option of attenuated total reflection (ATR) (Perkin Elmer, Waltham, MA, USA). Spectra were collected at eight scans per spectrum and 2 cm^−1^ resolution in the range of 650–4000 cm^−1^ at 25 °C.

### 4.3. High-Resolution Mass Spectrometry (HR-MS)

HR-MS analyses were performed on a Waters Xevo G2 Q-TOF mass spectrometer (Waters Corporation, Milford, MA, USA) equipped with an electrospray ionization (ESI) source operating in positive ion modes. Full-scan MS data were collected from 100–5000 Da in positive ion mode with a scan time of 0.1 s. To ensure accurate mass measurements, data were collected in centroid mode, and the mass was corrected during acquisition using leucine enkephalin solution as an external reference (Lock-Spray^TM^, Waters Corporation, Milford, MA, USA), which generated a reference ion at *m*/*z* 556.2771 Da ([M+H]^+^) in the positive ESI mode. The accurate mass and composition for the molecular ion adducts were calculated using the MassLynx 4.1 software (Waters) incorporated with the instrument.

### 4.4. UV–Vis Spectroscopy

UV–Vis spectroscopy (Evolution 300 Spectrophotometer, Thermo Scientific, Waltham, MA, USA) was performed to determine the T_CP_ of the star-comb polymers and their linear counterpart. The polymers were dissolved in distilled water at concentrations of 1 mg/mL, and 10 mg/mL. T_CP_ of the polymer solutions was determined by a heating–cooling–heating cycle. The polymer solutions were heated from 25 °C to 90 °C, then cooled to 25 °C, and finally heated again to 90 °C at a rate of 2 °C/min. The transmittance of the polymer solutions was measured at 542 nm. The T_CP_ was defined as the temperature at which an extremum of the first derivation is located by differentiating the transmittance vs. temperature curve.

### 4.5. Differential Scanning Calorimetry (DSC)

Thermal analyses were performed using a Mettler Toledo DSC 3 differential scanning calorimeter equipped with an XS105DU analytical balance (Greifensee, Switzerland). A total of ~1.5 mg (4.34 mg for SCP2) of the sample was used for measurements. The samples were positioned in 40 μL aluminum standard crucibles with a lid and a pin. Samples were tested in temperatures ranging from −20 °C to 280 °C (0–230 °C for SCP2) at a heating rate of 10 °C/min.

### 4.6. Size-Exclusion Chromatography (SEC)

Molecular weights (M_n_, SEC) and dispersity indices (Ð) were determined by a size-exclusion chromatograph (Ultimate 3000, Waltham, MA, USA) equipped with an isocratic pump, autosampler, degasser, thermostatic box for columns, and differential refractometer RefractoMax 521 Detector and DAWN^®^ (Waltham, MA, USA), and a multi-angle laser light scattering (MALLS) detector. ASTRA 7.3.2.17 data analysis software was used for data collecting and processing. The refractive index detection (RID) calculated molecular weight was based on calibration using linear polystyrene standards (M_p_ = 580–3,000,000 g/mol). A pre-column guard 5 μm 50 × 7.5 mm and double PLGel 5 μm MIXED-C and MIXED-D 300 × 7.5 mm column were used for separation. The measurements were carried out in THF (high-performance liquid chromatography (HPLC) grade) with the solvent at 35 °C with a flow rate of 1 mL/min.

### 4.7. Atomic Force Microscopy (AFM)

The analysis of topography was conducted using a BioScope Catalyst atomic force microscope from Veeco/Digital Instruments, which is equipped with a NanoScope V controller. The preparation of samples involved the following steps. First, a predetermined concentration (0.01 mg mL^−1^ and 0.001 mg mL^−1^) of the polymer solution under study was prepared in water. A volume of 5 μL of this solution was then dropped onto discharged mica and allowed to dry at room temperature. The BioScope Catalyst from Bruker was used to perform measurements in contact mode. A ScanAsyst-Air cantilever with a spring constant of 0.4 N m^−1^ was utilized for star-comb polymer SCP2 measurements, and a ScanAsyst-Fluid cantilever with a spring constant of 0.7 N m^−1^ was utilized for SCP3 and mikto-arm star-comb polymer MSCP1. The scanning speed was 0.65 μm/s, and the scan resolution was 512 × 512. All images were captured under ambient conditions. For the analysis of data, NanoScope version 1.80 and ImageJ Fiji, an open source software (1.54d) were employed.

## 5. Synthetic Methods and Procedures

### 5.1. Synthesis of Bromoisobutyryl-Functionalized CDs (8-Br-HP-β-CD)

HP-β-CD (2 g, 1.465 mmol) was placed in the jacketed reaction vessel and dissolved with an anhydrous NMP (20.6 mL) at 0 °C, resulting in a clear solution. TEA (1.305 g, 1.89 mmol) (deacid reagent) and a small amount (48 mg, 0.392 mmol) of DMAP (catalyst) were added to the solution. BiBB (2.695 g, 11.72 mmol) was diluted with anhydrous NMP (7.33 mL). The resulting solution was added dropwise to the HP-β-CD solution under an inert atmosphere (Ar). The mixture was stirred at 0 °C for 2 h, and then at room temperature for 24 h. The mixture containing the crude product was filtered to remove the triethylammonium bromide byproduct. The residual brown solution was precipitated in 800 cm^3^ water. Then, it was filtered under vacuum, and the white precipitate was washed with 2 L of water. Subsequently, the filter with the precipitate was left for 20 h to dry. Dichloromethane (20 mL) was used to dissolve the crude white solid, and the solution was recrystallized in cold n-hexane. The isolated white product (1.522 g, yield 40.63%) was dried under vacuum and kept in a sealed vial until required. The structure of 8-Br-HP-β-CD was confirmed by spectroscopic (^1^H NMR and ^13^C NMR (DMSO- d6, 600 MHz)) analyses. HR-MS (time of flight (TOF-ESI)) was calculated for 8-Br-HP-β-CD [M+K]^+^ 2289 (Supplementary Figure S1).

### 5.2. Synthesis of Star-Comb Polymers (Example for OEOMA300, 600:1 Molar Ratio to Initiator)

Star-comb polymer synthesis was performed by enzyme-assisted ATRP in the presence of air in phosphate-buffered saline (PBS), pH 7.4 at 45 °C for 2 h. The reaction was carried out in a glass vial, and the volume of the reaction mixture was 10 mL. First, OEOMA300 monomer (0.5 mL, 1.73 mmol) was added to the reaction vial. Then, glucose (0.18 g, 1 mmol) and GOx (0.0016 g, 10 nmol) were added to the vial. GOx catalyzed the oxidation of β-D-glucose to β-D-glucono-1,5-lactone and hydrogen peroxide (H_2_O_2_) using molecular oxygen as the electron acceptor. Subsequently, the previously synthesized initiator 8-HP-β-CD (7.39 mg, 2.89 µmol) (2-Hydroxyethyl 2-bromoisobutyrate for linear polymer CP (0.838 μL, 5.78 μmol)) was introduced (as a solid, entries SCP4, SCP6, SCP8, and SCP1-SCP3 or dissolved in 1.5 mL of methanol, entries SCP5, SCP7, and SCP9). Then, VA-044 (0.84 mg, 2.6 μmol) and CuCl_2_/TPMA (4.66 μL, 0.173 μmol CuCl_2_ and 0.519 μmol TPMA) (1:3 molar ratio in DMF, CuCl_2_ 5 mg/mL) were added. VA-044 was used as the reducing agent. CuCl_2_ was used as a catalyst, and TPMA was used as a ligand. Sodium pyruvate (55.02 mg, 0.5 mol) was added sequentially, followed by 9.5 mL of PBS (8 mL for entries with initiator dissolved in methanol). Sodium pyruvate eliminates H_2_O_2_ from the reaction mixture. The whole mixture was then thoroughly mixed and placed in an oil bath. After the reaction, 20 mL of methanol was added to the reaction mixture, and the reaction mixture was concentrated using a rotary evaporator to get rid of the water and methanol. Subsequently, 20 mL of THF was added to the residual reaction mixture to dissolve the polymer, which was then passed through a neutral aluminum oxide (Al_2_O_3_) column to get rid of the catalyst. The polymer was then precipitated three times in cyclohexane to dispose of the monomer and dried under a vacuum. The structures of the synthesized polymers were confirmed by ^1^H NMR spectra. Number average molecular weights (M_n, NMR_) were calculated based on ^1^H NMR spectra recorded for reaction mixtures. First, the conversion of monomer was calculated based on signals’ integrals from the vinyl group in unreacted monomer (average calculated from two signals at 6.04–6.01 ppm and 5.62–5.59 ppm) and methyl group from repeating units in the polymer (signals at 1.21–0.50 ppm). The degree of polymerization (DP) was determined by multiplying the monomer conversion by the initial ratio of monomer to initiator. DP per arm was determined by dividing the total DP by 8 referring to the number of initiation sites of the initiator. M_n_ from NMR was calculated by multiplying the DP value times the molar weight of the monomer.

### 5.3. Synthesis of Mikto-Arm Star-Comb Polymers Using ROP

The Schlenk reactor was subjected to a silanization process 24 h before carrying out the ROP. This pre-treatment involved the introduction of a 6% *v*/*v* solution of trimethylchlorosilane [(CH_3_)_3_SiCl] in toluene into the reactor. Following the required time, the silanizing solution was decanted, and the reactor underwent a drying process under vacuum conditions at a temperature of 140 °C for a duration of two hours. Subsequently, a macroinitiator (0.2 g, 2.17 μmol), solubilized in toluene (1 mL), along with the ε-caprolactone (ε-CL, 31.3 mL, 283 μmol), was introduced into the silanized Schlenk reactor. The reaction mixture was purged with inert gas. Thereafter, the catalyst, tin(II) 2-ethylhexanoate [Sn(Oct)_2_] (0.7 μL, 2.17 μmol), was incorporated into the reaction mixture. The Schlenk reactor was then positioned within an oil bath calibrated to a temperature of 100 °C. Upon completion of the reaction, the reactor was extricated from the oil bath and subjected to a cooling period of 15 min. The resultant product was isolated through a dual precipitation process using n-heptane as the precipitating agent. The isolated product was then subjected to a drying process at ambient conditions under a vacuum until a constant mass was achieved. ^1^H NMR spectra analysis confirmed the final structure of MSCP1.

## 6. Conclusions

In this work, star-comb polymers with OEOMA arms and CD-based cores were synthesized by enzymatically assisted ATRP in the presence of air. The polymers were characterized by ATR-FTIR, ^1^H NMR, ^13^C NMR, UV–Vis, and SEC/MALLS analyses. The thermosensitivity of the polymers was evaluated by measuring their cloud point temperatures in water. The well-defined star-comb POEOMA300s obtained using a solution of initiator in methanol were characterized by narrow molecular weight distributions (M_w_/M_n_ < 1.35). The various lengths of arms and cores based on CD can be useful for future applications as a polymeric absorbent of metals in wastewater treatment and their subsequent recovery, in controlled drug release systems, biomedicine, cosmetics, the food industry, and in agriculture. Due to the relatively high T_CP_ of OEOMA300-based polymers, they are not suitable for use as potential drug delivery systems throughout the body. However, they can be applied topically, using methods like laser heating above the T_CP_. Nevertheless, they are suitable for potential industrial applications, particularly in the removal of pollutants, such as heavy metals, from water. Thus, these polymers contribute to an environmentally friendly approach, providing a novel strategy for the synthesis of well-defined star-comb polymers following the principles of green chemistry.

## Figures and Tables

**Table 1:** Characteristics of the obtained polymers.

Entry	Reaction Conditions	OEOMA Conversion [%]	DP_OEOMA_	DP per Arm	M_n NMR_ [g/mol]	M_n, SEC_ [g/mol]	M_w, MALLS_ [g/mol]	Ð _SEC_
SCP1	[OEOMA500]_0_:[8-Br-HP-β-CD]_0_:[VA-044]_0_:[CuCl_2_]_0_:[TPMA]_0_ = 600:1:0.9:0.06:0.18	94%	564	71	282,000	65,900	325,500	1.38
SCP2	[OEOMA500]_0_:[8-Br-HP-β-CD]_0_:[VA-044]_0_:[CuCl_2_]_0_:[TPMA]_0_ = 400:1:0.9:0.06:0.18	95%	380	48	190,000	175,700	214,400	1.26
SCP3	[OEOMA500]_0_:[8-Br-HP-β-CD]_0_:[VA-044]_0_:[CuCl_2_]_0_:[TPMA]_0_ = 200:1:0.9:0.06:0.18	91%	182	23	91,000	103,100	111,900	1.23
CP	[OEOMA300]_0_:[HO-EBiB]_0_:[VA-044]_0_:[CuCl_2_]_0_:[TPMA]_0_ = 600:1:0.9:0.06:0.18	81%	486	486	145,800	76,000	124,900	1.61
SCP4	[OEOMA300]_0_:[8-Br-HP-β-CD]_0_:[VA-044]_0_:[CuCl_2_]_0_:[TPMA]_0_ = 600:1:0.9:0.06:0.18	89%	534	67	160,200	380,700	-	1.75
SCP5	[OEOMA300]_0_:[8-Br-HP-β-CD]_0_:[VA-044]_0_:[CuCl_2_]_0_:[TPMA]_0_ = 600:1:0.9:0.06:0.18 (+1.5 mL MeOH with initiator)	65%	390	49	117,000	686,00	98,500	1.34
SCP6	[OEOMA300]_0_:[8-Br-HP-β-CD]_0_:[VA-044]_0_:[CuCl_2_]_0_:[TPMA]_0_ = 00:1:0.9:0.06:0.18	86%	344	43	103,200	294,000	-	1.44
SCP7	[OEOMA300]_0_:[8-Br-HP-β-CD]_0_:[VA-044]_0_:[CuCl_2_]_0_:[TPMA]_0_ = 400:1:0.9:0.06:0.18 (+ 1.5 mL MeOH with initiator)	68%	272	34	81,600	56,800	76,800	1.26
SCP8	[OEOMA300]_0_:[8-Br-HP-β-CD]_0_:[VA-044]_0_:[CuCl_2_]_0_:[TPMA]_0_ = 200:1:0.9:0.06:0.18	89%	178	22	53,400	150,600	-	1.69
SCP9	[OEOMA300]_0_:[8-Br-HP-β-CD]_0_:[VA-044]_0_:[CuCl_2_]_0_:[TPMA]_0_ = 200:1:0.9:0.06:0.18 (+ 1.5 mL MeOH with initiator)	79%	158	20	47,400	34,400	43,300	1.27

Where: M_n, NMR_—number average molecular weight based on ^1^H NMR; M_n_, _SEC_—number average molecular weight based on SEC; Ð—dispersity index based on SEC, yield was calculated by dividing the weight of the dry product from the weight of the substrate, “-”—not determined.

## Data Availability

Data are contained within the article and supplementary materials.

## Supplementary Materials

The following supporting information can be downloaded at: https://www.mdpi.com/article/10.3390/molecules29010055/s1, Figure S1. HR-MS spectra of the obtained 8-Br-HP-β-CD initiator; Figure S2. IR spectra of HP-β-CD and 8-Br-HP-β-CD initiator; Figure S3. 13C NMR (600 MHz, DMSO) spectra of 8Br-HP-β-CD; Figure S4. 2 DSC thermograms for HP-β-CD and 8-Br-HP-β-CD; Figure S5. SEC traces of MSCP1 and SCP3; Figure S6. AFM images of obtained star-comb-polymer SCP8 at 0.001 mg/mL; Figure S7. DSC thermograms for SCP2; Table S1. Characteristics of the obtained mikto-arm polymers. Supplementary data associated with this article can be found in the online version.
