# Supplementary material for: Eco-Conscious Approach to Thermoresponsive Star-Comb and Mikto-Arm Polymers via Enzymatically Assisted Atom Transfer Radical Polymerization Followed by Ring-Opening Polymerization
Source: Molecules. 2023 Dec 21;29(1):55. doi: 10.3390/molecules29010055 (PMC10779862; doi:10.3390/molecules29010055)
Supplement: Supplementary file 1 [file molecules-29-00055-s001.zip › molecules-2741546-supplementary.pdf]

The Supplementary Information

Eco -Conscious Approach to Thermoresponsive Star-Comb and Mikto-Arm Polymers via Enzymatically Assisted Atom Transfer Radical Polymerization Followed by Ring-Opening Polymerization

Tomasz Fronczyk[1], Anna Mielańczyk[1], Olesya Klymenko[2], Karol Erfurt[3], Dorota Neugebauer[1]

[1]Department of Physical Chemistry and Technology of Polymers, Faculty of Chemistry, Silesian University of Technology, M. Strzody 9 Street, 44-100 Gliwice, Poland

[2]Department of Histology and Cell Pathology, School of Medicine with the Division of Dentistry in Zabrze, Medical University of Silesia, 41-808, Zabrze, Poland

[3]Department of Organic Chemical Technology and Petrochemistry, Faculty of Chemistry, Silesian University of Technology, B. Krzywoustego 4, 44-100 Gliwice, Poland

* Author to whom correspondence should be addressed: *anna.mielanczyk@polsl.pl*

Table of content

1. Fig. S1. HR-MS spectra of the obtained 8-Br-HP-β-CD initiator. 2
2. Fig. S2. IR spectra of HP-β-CD and 8-Br-HP-β-CD initiator. 2
3. Fig. S3. ^{13}C NMR (600 MHz, DMSO) spectra of 8-Br-HP-β-CD. 3
4. Fig. S4. 2 DSC thermograms for HP-β-CD and 8-Br-HP-β-CD. 3
5. Fig. S5. SEC traces of MSCP1 and SCP3. 4
6. Fig. S6. AFM images of obtained star-comb-polymer SCP8 at 0.001 mg/mL. 4
7. Fig. S7. DSC thermograms for SCP2. 5
8. Table S1. Characteristics of the obtained mikto-arm polymers. 5

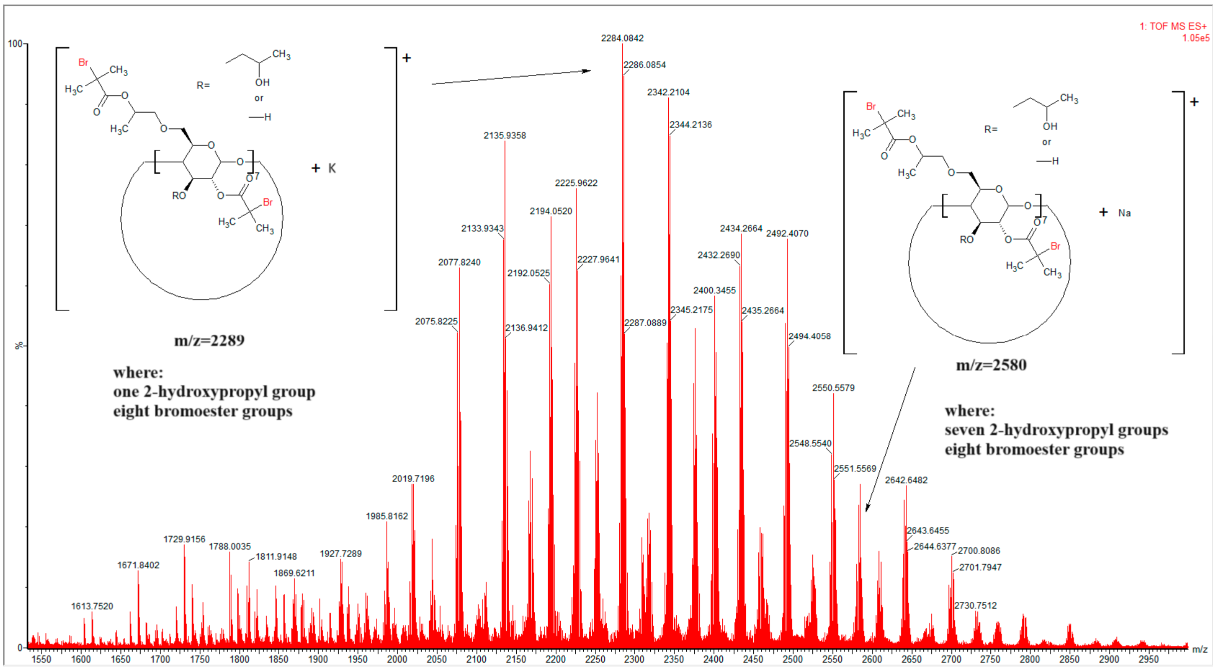

Fig. S1. HR-MS spectra of obtained 8-Br-HP-β-CD initiator.

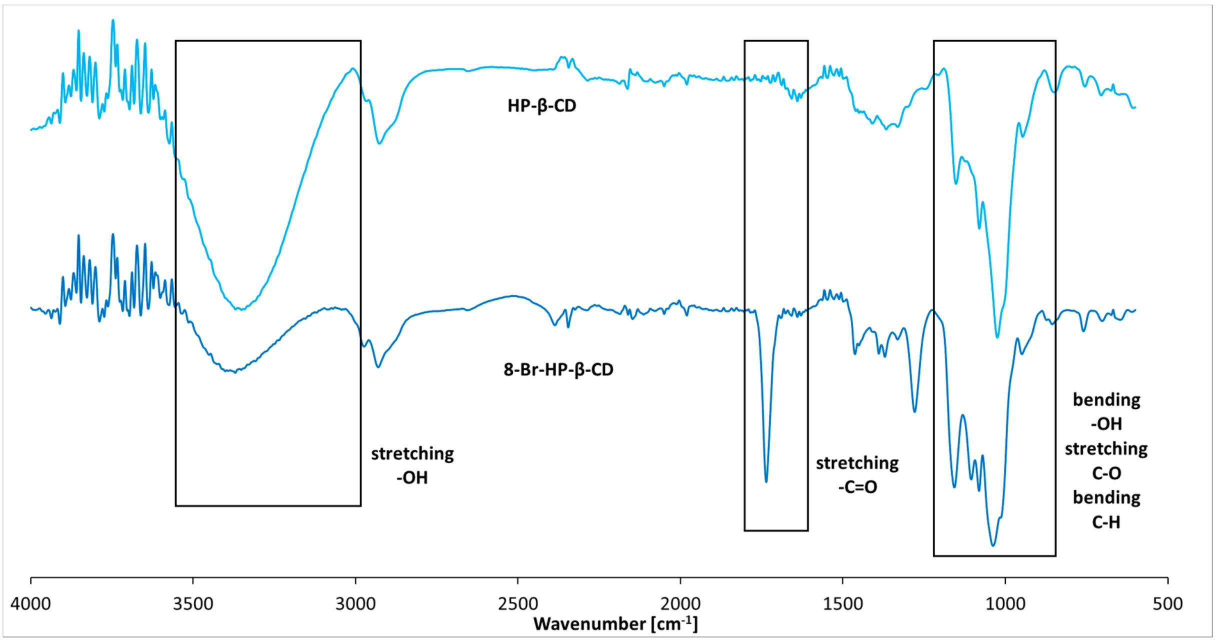

Fig. S2. IR spectra of HP-β-CD and 8-Br-HP-β-CD initiator.

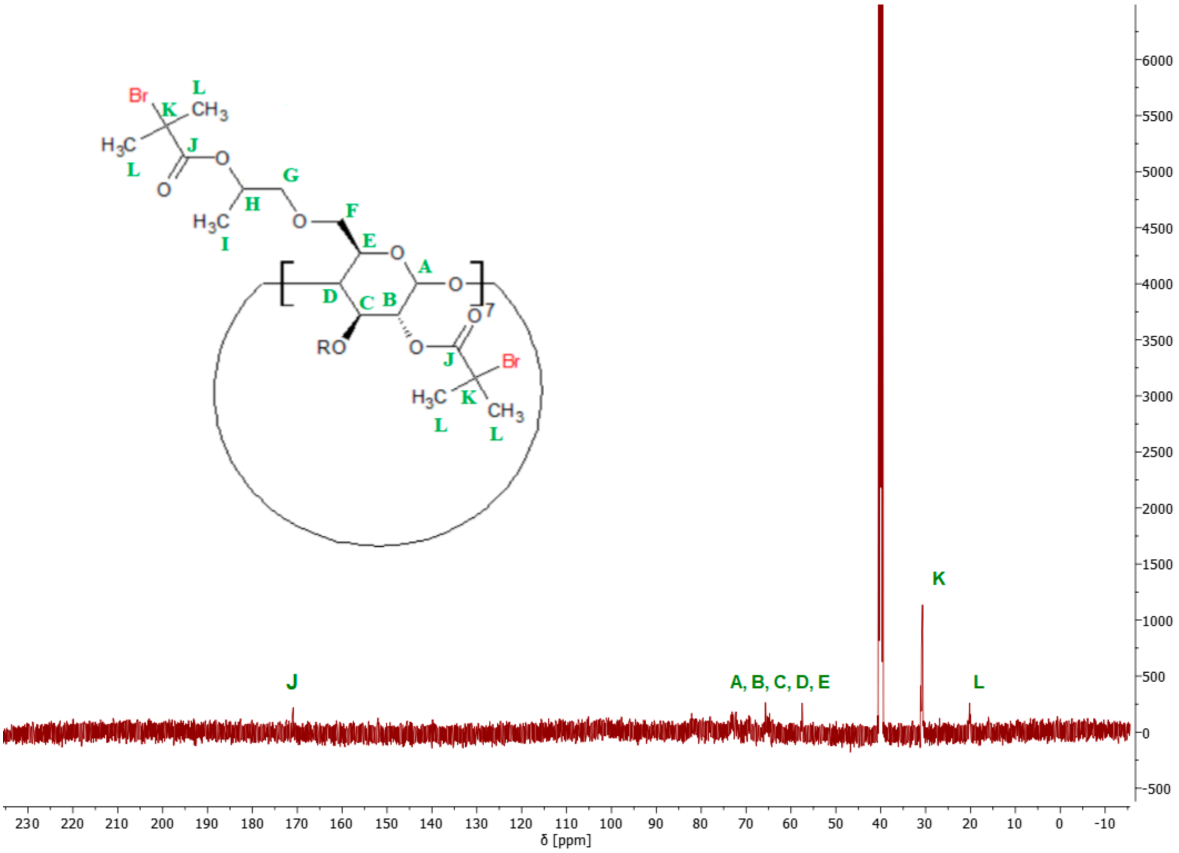

Fig. S3. ^{13}C NMR (600 MHz, DMSO) spectra of 8-Br-HP-β-CD.

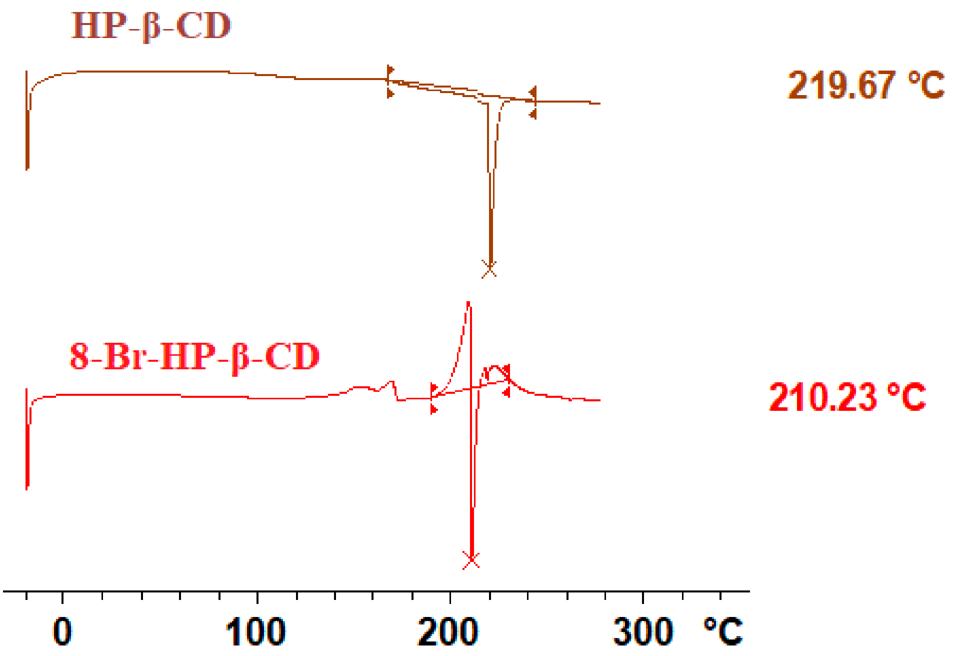

Fig. S4. DSC thermograms for HP-β-CD and 8-Br-HP-β-CD.

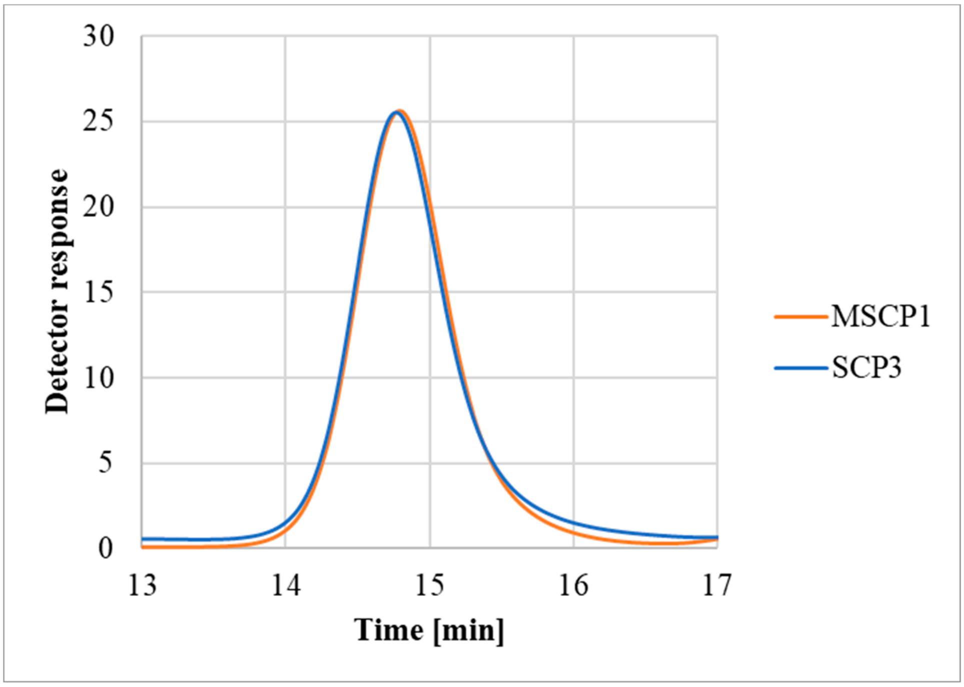

Fig. S5. SEC traces of MSCP1 and SCP3.

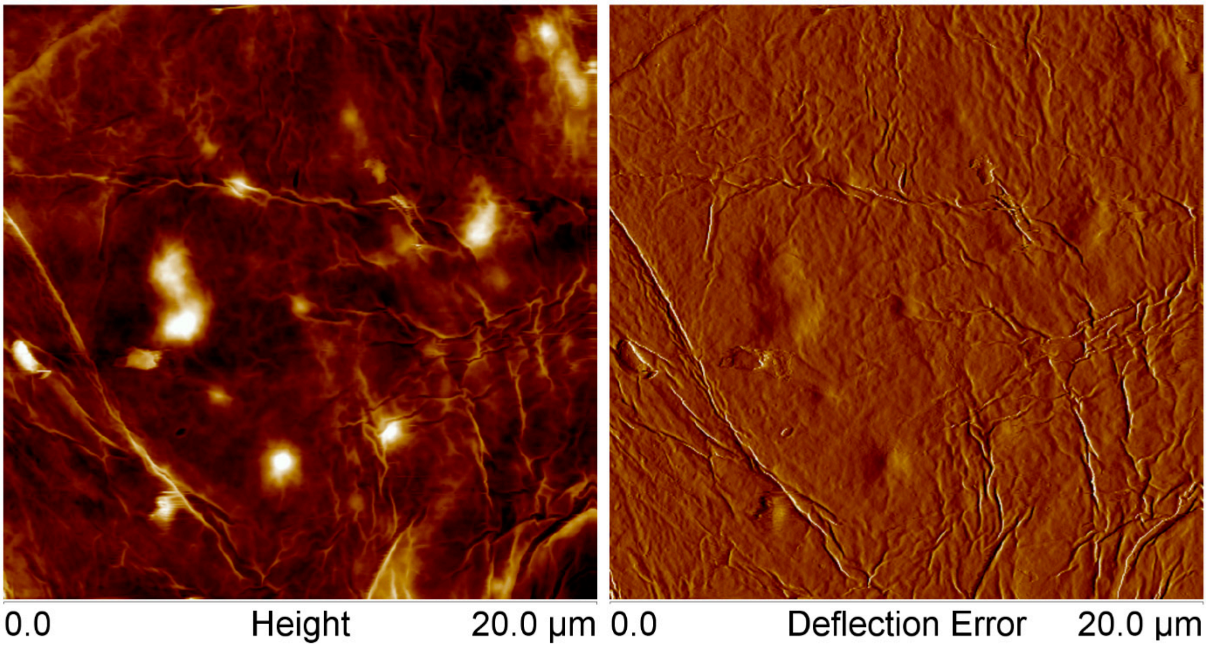

Fig. S6. AFM images of the obtained star-comb polymer SCP2 at 0.001 mg/mL.

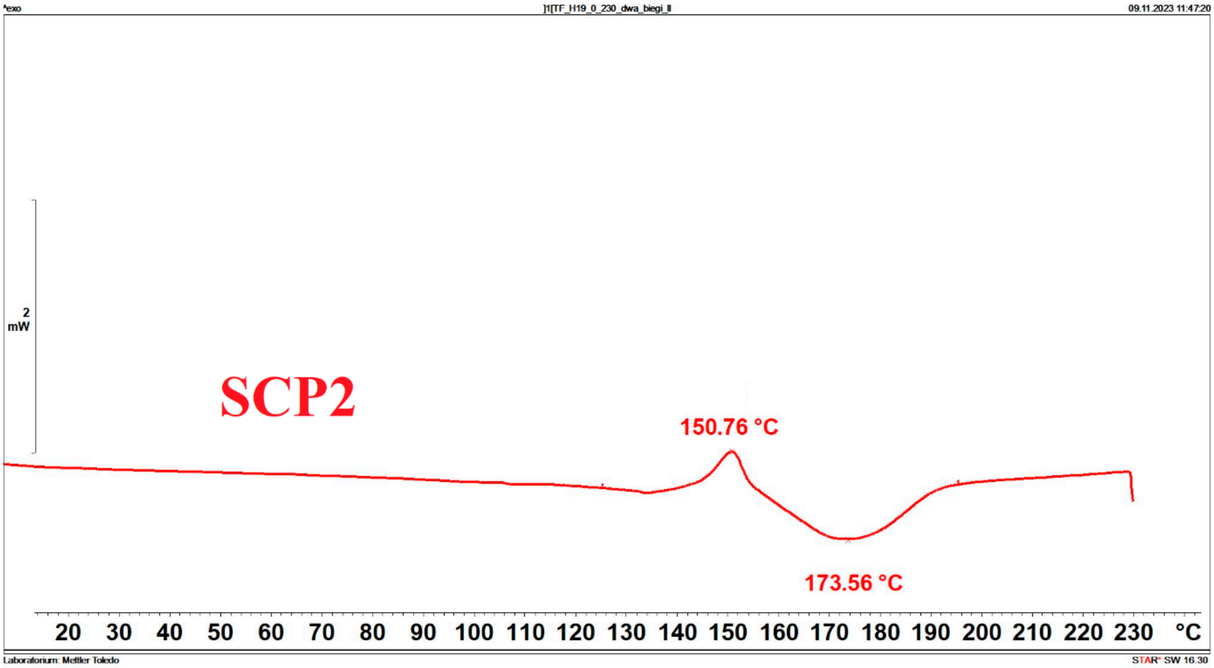

Fig. S7. DSC thermograms for SCP2.

Table S1. Characteristics of the obtained mikto-arm polymers.

Entry	Reaction Conditions	ε-CL conversion [%]	$DP_{polyester}$	Mole fraction $_{ester}$ [%]	$M_{n\ NMR}$ [g/mol]	$M_{n,\ SEC}$ [g/mol]	$M_{w,\ MALLS}$ [g/mol]	$Đ_{SEC}$
MSCP1	$[SCP3]_0$:[ε-CL]$_0$: $[Sn(Oct)_2]_0$ =1:130:1	36	47	21	97,300	51,300	96,200	1.31
MSCP2	$[SCP9]_0$:[ε-CL]$_0$: $[Sn(Oct)_2]_0$ =1:130:1	69	90	36	55,800	29,100	47,200	1.20
